# Analysis of modified plug-in electric vehicle charger controller with grid support functionalities

**DOI:** 10.1371/journal.pone.0262365

**Published:** 2022-01-27

**Authors:** Selvakumar R. B., Vivekanandan C.

**Affiliations:** Department of Electrical and Electronics Engineering, Dr.N.G.P. Institute of Technology, Coimbatore, Tamilnadu, India; University of Science and Technology of China, CHINA

## Abstract

Power quality issues, which are mainly due to power electronic devices that are invariably used not only in domestic sector but also industries, still persist despite various mitigation strategies. The slow but steady invasion of Electric vehicles or Plug-in Electric Vehicles (PEVs) in recent years, in the automobile sector, adds woes to the power quality issues further. Majority of the charging systems presently available for charging PEVs are unidirectional and so supports Grid to Vehicle (G2V) mode only as the bidirectional integration of those vehicles into the grid is still a big challenge. However, Vehicle to Grid (V2G) support mode also deserves an equal importance as the PEV charger with V2G mode of operation is capable of supporting grid functionalities also, on need basis, which largely depends on the power circuit topology and controller topology it uses. Hence, in this work an improved controller topology has been designed and developed to alleviate the burdens on the grid. Support for active power demand, voltage swell and sag mitigation, in addition to catering its prime objective of charging the batteries are focused. A Second Order Generalized Integrator Phase Locked Loop (SOGI-PLL) based controller has been developed and implemented in the proposed work to improve the transient response, apart from controlling the steady-state oscillations of the grid to which it is connected to. A single phase non-isolated bidirectional PEV charger with proposed control topology has been simulated in MATLAB-Simulink for vehicle support and grid support mode of operations. The simulation proves the satisfactory operation of the proposed charger in the four quarters of active power and reactive power (PQ) plane, thus complies the design objectives of bidirectional power flow. The results obtained from the simulation show improved performance in terms of DC link voltage overshoot, steady-state oscillations, overall efficiency, voltage and current Total Harmonic Distortions (THD)

## 1. Introduction

Plug-in Electric Vehicles are slowly transforming the global transportation sector and the rapid advancement in PEV technology increases their popularity. The necessity of PEVs in automobile sector arises due to the emission of unsolicited gases while using fossil fuel based vehicles, the cause of inescapable environmental pollution. PEVs yield promising solution while addressing the above issues [1]. The vision for the smart electric grid to transition towards more penetration of distributed energy resources and the usage of PEVs and energy storage encourage the extensive inclusion of renewable energy sources and PEVs into the grid. This induces great challenges to grid operation and control due to their intermittent nature. The utilities need to pay more attention on grid regulation, peak load shaving, spinning reserve, load leveling, reactive power compensation and power quality services [2]. However, the Vehicle to Grid (V2G) enabled PEVs can act as prosumer (Producer/Consumer) in the modern smart grid environment with their energy storage system (ESS) and offer the above services in short duration to the grid [3–8]. In this context, the concept of V2G is firstly proposed by Kempton and Letendre [9]. Different operating modes for PEVs are discussed in [10, 11]. The detailed review of PEV charger types and charging power levels are found in [12, 13]. Due to simple structure, high efficiency and low cost, the single-phase non-isolated V2G enabled PEV chargers find their place in low power slow-charging and grid support applications. Nonetheless, the complexity in achieving the fast dynamic response during G2V and V2G modes of operation, lowering the power quality impact on grid and further improvement in efficiency necessitates the enhanced variants of such PEV charger in terms of circuit and control topology.

Literatures that addressed different single phase PEV Charger topologies, have paid more attention on G2V mode of operation alone [14–16]. The major reason is the importance on fast charging capability that is comparable to conventional internal combustion engine vehicles in terms of refueling speed. So, the issues during power flow from grid to battery to charge the latter only are discussed. Contrarily, even if the V2G mode of operation is addressed in a fewer literatures, it is observed that the performance of the PEV charger during this mode is relatively inferior and unable to comply with standard specifications [17, 18].

The grid synchronization of bidirectional PEV charger is a crucial problem as the grid voltage information need to be measured and controlled. A phase-locked loop (PLL) is a popular closed-loop method for the above said problem due to its simplicity, robustness and effectiveness. However, the dynamics of the system is affected by the performance of simple PLL under distorted conditions. Therefore, the grid connected PEV charger necessitates the selection of proper PLL to prove their superiority. The performance of various types of PLL techniques are discussed and compared in [19]. Comparison results show that the SOGI-PLL stands strong in terms of high disturbance rejection, filtering capacity, immune to noise, fast dynamic response and harmonic extraction. Therefore, to circumvent the issues with a simple PLL used in the present works, the SOGI-PLL is used in the proposed work [20–24].

Anjeet Verma et al. [25] have proposed different controllers to utilize solar energy for PEV charging. Mithat et al. [26] have discussed the limitations of bidirectional charger during reactive power compensation. The grid side filters implemented in existing chargers is replaced by an LCL filters in the proposed work, since, LCL filters have better control in reducing inrush current at lower frequencies and improves THD marginally [27, 28]. Also, IGBT is used in lieu of MOSFETs in the proposed PEV charger, as the former incur relatively lesser conduction losses.

Further, from the available literature it is observed that the higher importance is given to reactive power compensation, and injection of active power, back to the grid on demand, is seldom discussed [17]. Hence, a charger that adaptively adjusts both reactive power compensation and active power injectionis proposed in this work.

**Fig 1:**
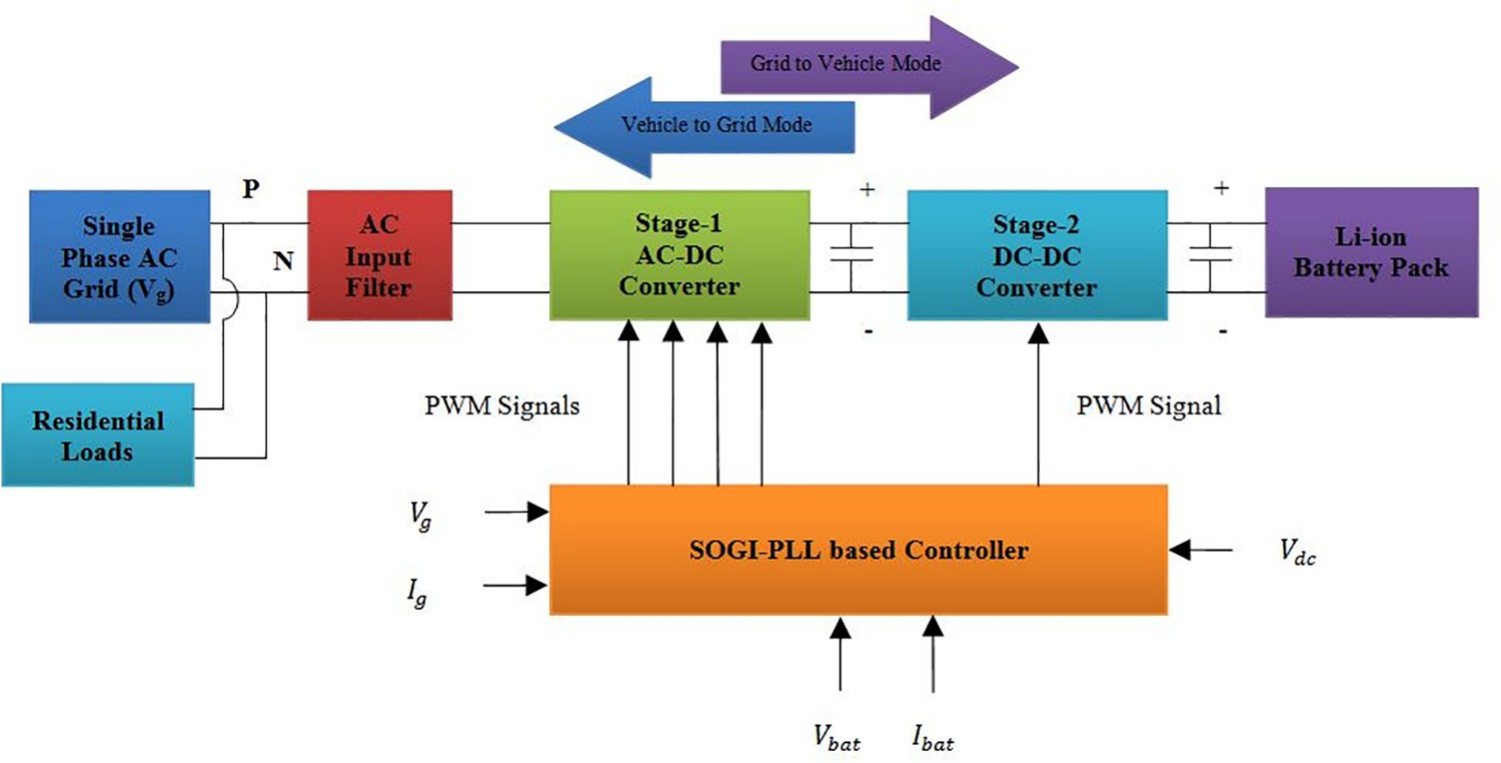
Single phase non-isolated bidirectional charger for PEVs.

There are two converter stages in the proposed circuit topology as depicted in Fig 1. The first stage is a four-quadrant bidirectional converter that operates as rectifier in vehicle support mode and operates as inverter in grid support mode. Based on the specified active power demand (*P*_*c*_) and reactive power demand (*Q*_*c*_) inputs from smart grid environment, the four quarters of active-reactive power (PQ) plane is covered through the converter operation in eight different modes [29]. The second stage is a two-quadrant DC-DC converter that operates as buck converter during vehicle support mode and operates as boost converter in grid support mode [30]. Functioning of both the converters discussed above are controlled by a modified two stage cascaded controller. The SOGI-PLL used in the first stage of the controller tracks the phase-angle of grid voltage and generates a signal of reference for charger current. The second stage of the controller ensures constant current charging mode of PEV battery-pack by regulating the instantaneous battery current. The battery reference current is generated by using variable dc-link voltage control.

Different types of battery models like equivalent circuit models, electrochemical models and empirical models has been widely investigated by the researchers for system simulation and controller design in [31–35]. The equivalent circuit model added with additional resistor-capacitor (RC) networks to improve accuracy results in reduced computational efficiency and also need more detailed experimental data to determine its model parameters. The complicated chemical reaction and transport equations restricts the use of Multi-physics Electrochemical models for grid applications though they are well developed. In [36], the generic MATLAB battery models are analyzed in detail and a modified dynamic model is proposed. Due to simplicity and relatively high accuracy, the generic models are widely employed in various applications. Therefore, the generic MATLAB battery model is used in the proposed system simulation [37].

Section II elaborates the system description of proposed single phase non-isolated bidirectional PEV charger. Section III emphases on the proposed charger controller details for constant current charging of PEV battery, active power control and reactive power control. Section IV presents the MATLAB-Simulink model and simulation conditions of the proposed charger controller. Section V examine the simulation results of the proposed charger controller. Section VI compares the simulation result of proposed charger controller with existing works.

## 2. System description of single phase non-isolated bidirectional PEV charger

**Fig 2:**
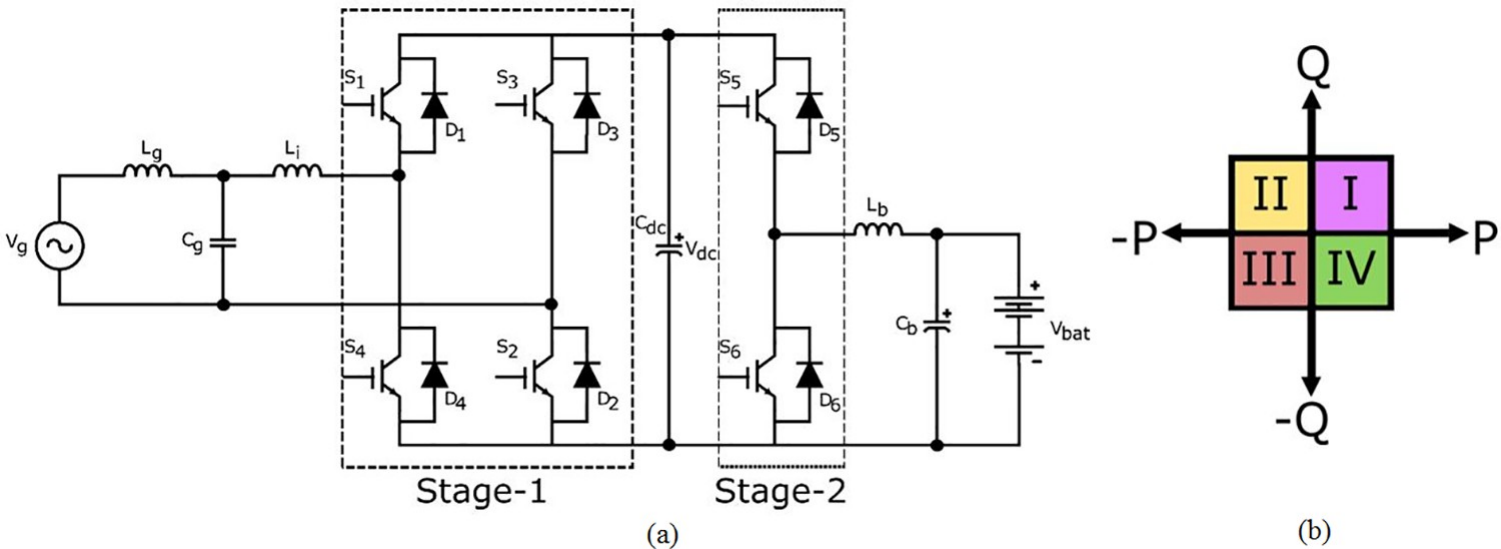
Non-isolated bidirectional PEV charger (a) Power circuit topology (b) Quadrant diagram of active-reactive power plane.

The schematic of the proposed non-isolated bidirectional PEV charger, with two power converter stages, is shown in Fig 2A. As shown in the figure, Stage-1 bidirectional full bridge converter has four controllable switches *S*_1_, *S*_2_, *S*_3_ and *S*_4_ with diodes *D*_1_, *D*_2_, *D*_3_ and *D*_4_ connected in anti-parallel. The arrangement ensures the power flow in two directions. The properly selected grid side inductor (*L*_*g*_), grid side capacitor (*C*_*g*_) and inverter side inductor (*L*_*i*_) filter curtails the current harmonics, introduced during vehicle support and grid support modes of operation [27, 28].

The stage-2 of power circuit is the modified form of unidirectional DC-DC converter. A controllable switch *S*_6_ and diode *D*_5_ are added with diode *D*_6_ and a controllable switch *S*_5_ respectively to form two directional DC-DC converter. There is an LC filter realized with battery side inductor (*L*_*b*_) and battery side capacitor (*C*_*b*_) at the output stageto minimize ripples in charging current. The DC-Link Capacitor (*C*_*dc*_) connects the two power converter stages of bidirectional PEV charger. The operating modes of Stage-1 converter is tabulated in Table 1.

**Table 1:** Operating modes of stage-1 converter.

Mode		Operation	Active Power, P	Reactive Power, Q
1	G2V	Charge-only	Positive	Zero
2	G2V	Charge-Inductive	Positive	Positive
3	G2V	Charge-Capacitive	Positive	Negative
4	-	Inductive-only	Zero	Positive
5	V2G	Discharge-only	Negative	Zero
6	V2G	Discharge-Inductive	Negative	Positive
7	V2G	Discharge-Capacitive	Negative	Negative
8	-	Capacitive-only	Zero	Negative

The stage-2 bidirectional DC-DC converter operates in Buck-mode during charger delivers power to vehicle battery pack, whereas it operates in Boost-mode during charger delivers active power to grid. The switching condition during different intervals of operation of the devices *S*_5_, *D*_5_, *S*_6_ and *D*_6_ are tabulated in Table 2.

**Table 2:** Switching conditions of stage-2 converter.

Buck / Boost	Interval	Condition of Switches			
		*S*_5_	*D*_5_	*S*_6_	*D*_6_
Buck (G2V)	Interval—1	Close	Open	Open	Close
	Interval—2	Open	Open	Open	Close
Boost (V2G)	Interval—3	Open	Open	Close	Open
	Interval—4	Open	Close	Open	Open

## 3. Controller for proposed charger

To comply with the design objectives of the proposed PEV charger, it is necessary to coordinate the operations of both stages of the aforesaid converter properly through the controller. The complete functionalities of the controller, at different stages of operations of the PEV charger are detailed below.

**Fig 3:**
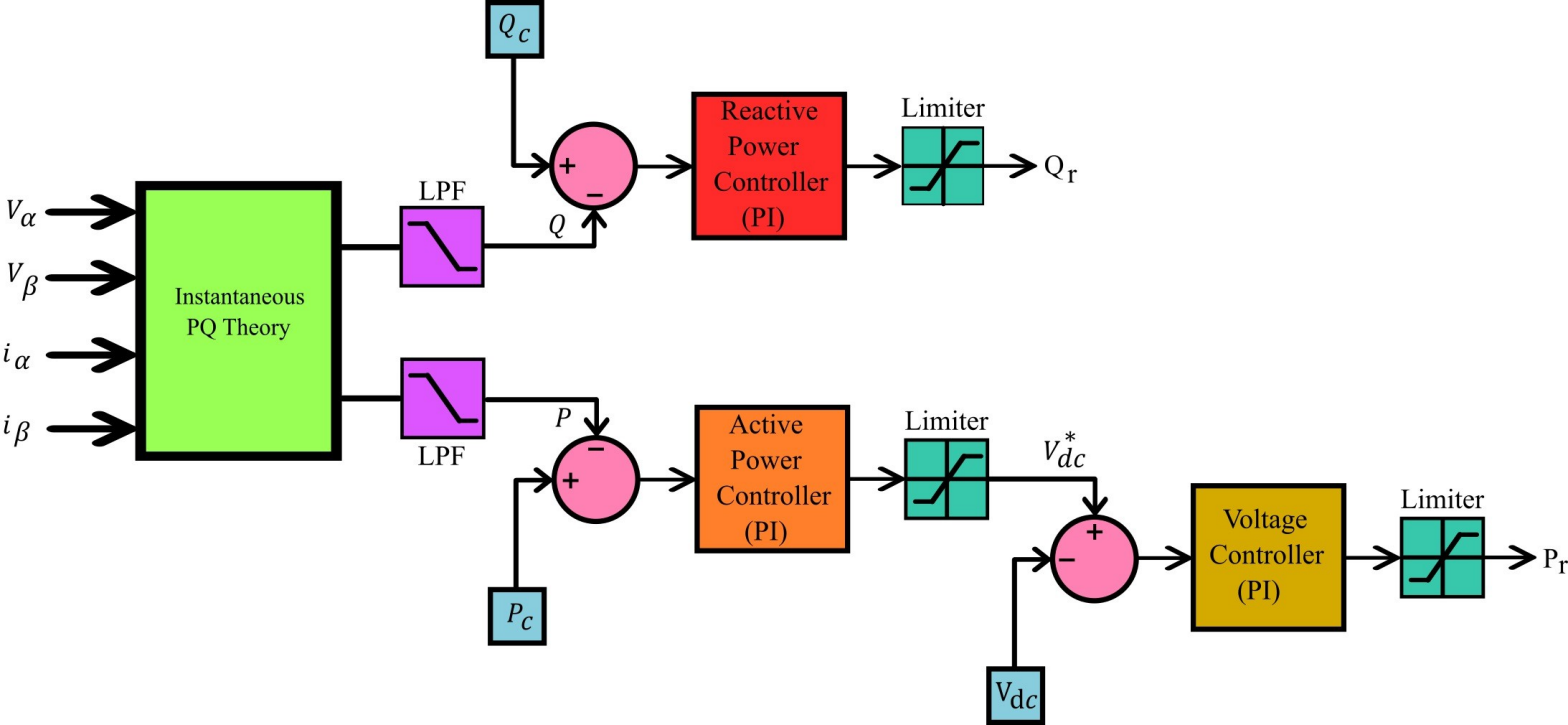
Controller subsection-1 for converter stage-1.

**Fig 4:**
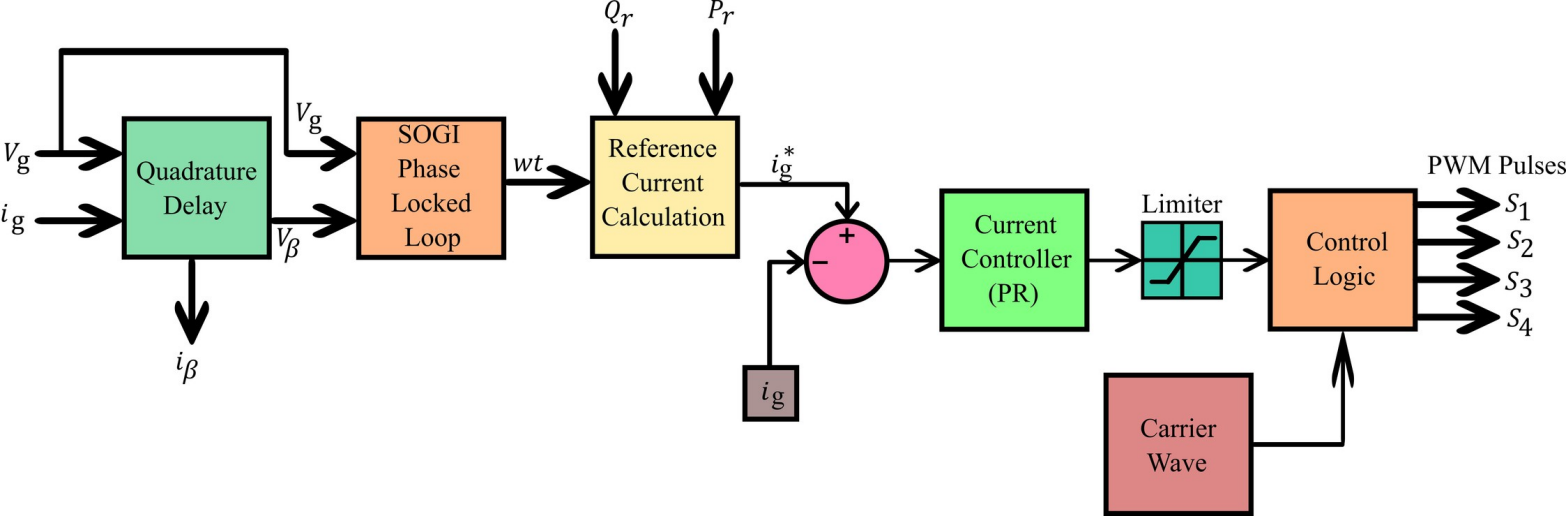
Controller subsection-2 for converter stage-1.

The integration of the converter stage-1, i.e. four-quadrant bidirectional converter, in to the grid is an adaptive task in view of the inevitable fluctuations in the grid [21]. Consequently, it is necessary to generate a reference signal for the parameters such as voltage, current and frequency continuously, for the aforesaid synchronization. The SOGI-PLL is provided with Grid voltage (*V*_*g*_) as input where it is split in to alpha component and beta component. The alpha-beta components are transferred to *dq* components from which the information about the grid voltage phase-angle (*ωt*) is derived and is available at the output of SOGI-PLL. The beta component of the grid current (*i*_*β*_) is obtained using delay function. There are two controller subsections for converter stage-1, wherein the purpose of the first section, shown in Fig 3, is to generate active power reference (*P*_*r*_) and reactive power reference (*Q*_*r*_) with the available information about alpha and beta parts of grid voltage (*V*_*α*_, *V*_*β*_) and current (*i*_*α*_, *i*_*β*_). The generated *P*_*r*_ and *Q*_*r*_, along with SOGI-PLL in second subsection of the controller, are used to generate the necessary Pulse Width Modulation (PWM) signals for the controllable switches *S*_1_ to *S*_4_ of converter Stage-1 as shown in Fig 4. The operation of the controller is as follows.

The controller subsections of converter Stage-1, depicted in Figs 3 and 4, are so designed that all the four quarters of PQ plane is covered through converter operation, by tracking demands raised for *P*_*c*_ and *Q*_*c*_ requirements. The grid active power (*P*) and reactive power (*Q*) are estimated using the instantaneous PQ theory. The difference between the estimated *P* and the *P*_*c*_ is fed to a pair of PI controllers. The first PI controller satisfies *P*_*c*_ by temporarily modifying the dc link voltage reference $\left(V_{dc}^{*}\right)$ and the second one tracks the $V_{dc}^{*}$ set by the first PI controller and produces active power reference signal *P*_*r*_. Similarly, the generation of reactive power reference signal *Q*_*r*_ is carried out, but with a single PI controller as shown in Fig 3. The output *wt* of the SOGI PLL, along with the generated signals *P*_*r*_ and *Q*_*r*_ insubsection-1 is used for calculating the charger current reference $\left(i_{g}^{*}\right)$. The error obtained from the continuous comparison of $i_{g}^{*}$ and actual grid current (*i*_*g*_) is fed to a Proportional and Resonant Current controller (PR Controller) [38]. The required PWM signals for the controllable switching devices of stage-1 converter is generated by utilizing the output of PR controller.

**Fig 5:**
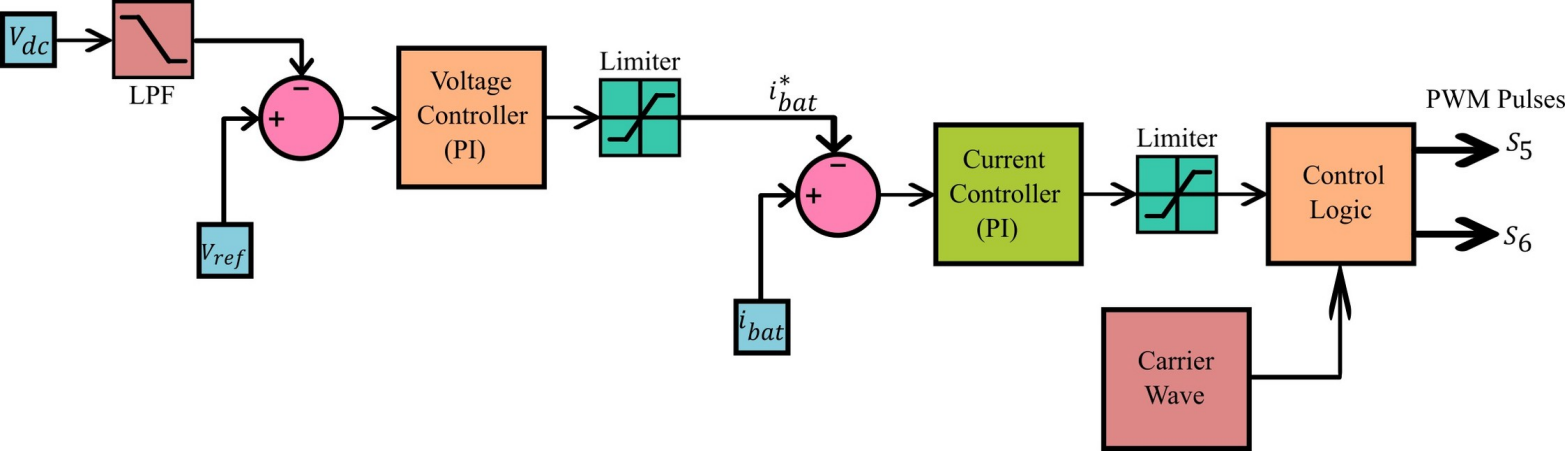
Controller for converter stage-2.

The suitable control of the switches in stage-2 bidirectional DC-DC converter ensures the realization of two-quadrant operation in which the voltage component is always positive whereas the current is bidirectional. In the forward direction i.e. during G2V mode, fast charging is ensured by maintaining constant charging current and in the reverse direction effective V2G operation is ensured by maintaining constant DC link voltage. The controller block diagram of this process is shown in Fig 5. During charging, the converter is in buck-mode, to match with battery voltage standards. The DC link voltage (*V*_*dc*_) and reference voltage (*V*_*ref*_) are compared using a comparator and the error signal is given as input to the voltage controller. The voltage controller generates required battery reference current $\left(i_{bat}^{*}\right)$. The suitable design of a current controller ensures the preferred constant current charging mode of operation. The output of the current controller depends on the difference between the present magnitude of the battery charging current (*i*_*bat*_) and $i_{bat}^{*}$. The PWM signal for the switches in converter stage-2 is generated using the duty cycle. The duty cycle is controlled by current controller output. In boost mode, it is evident that the current is in reverse direction i.e. from battery to DC link with rest of the configuration remains same.

## 4. Simulation of the proposed charger controller

**Fig 6:**
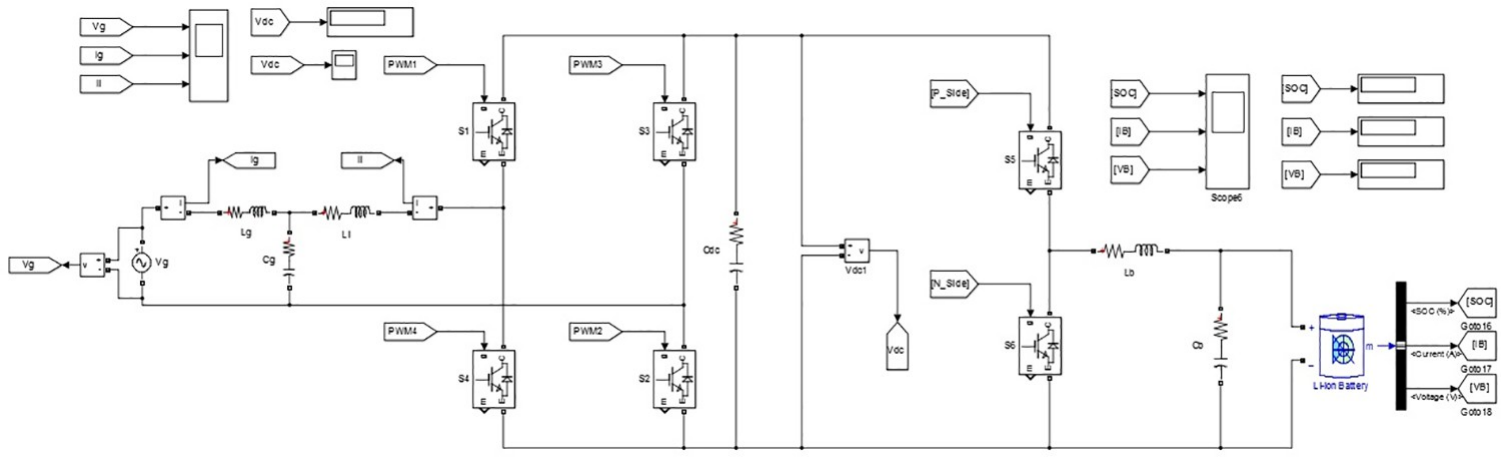
Simulation model of proposed PEV charger.

The proposed single phase non-isolated bidirectional PEV charger controller is simulated using MATLAB-Simulink to assess the performance of the former. Fig 6 depicts the schematic of the proposed charger realized using various fundamental blocks available in Simulink. The rating of the proposed charger is assumed to be 1.35 kW with a maximum charging current of 5.5 Amps at a charging voltage of 250 Volts. The switching device selected is IGBT available under Simscape library, with internal resistance 1 *m*Ω and snubber resistance 0.1 *M*Ω, for both the converter stages 1 and 2. The Lithium-ion battery type in generic MATLAB battery model is utilized for system simulation. The parameters chosen for simulation of proposed PEV charger are presented in Table 3. The battery voltage level in PEVs ranges between 200 and 400 V. Therefore battery nominal voltage of 240 V and DC link voltage of 340 V are chosen in this paper for testing [39].

**Table 3:** System parameters used for simulation.

Notation	System Parameter	Specification / Value
*P*_*ch*_	Charger Power rating	1.35 kW
*V*_*g*_	Grid voltage	230 *V*
*f*	Grid frequency	50 *Hz*
*L*_*g*_	Grid side inductance	5.9 *mH*
*L*_*i*_	Inverter side inductance	6.38 *mH*
*C*_*g*_	Grid side capacitance	4.12 μ*F*
*f*_*s*1_	AC-DC converter switching frequency	10 *kHz*
*V*_*dc*_	DC-link voltage	340 *V*
*C*_*dc*_	DC-link capacitance	2200 μ*F*, 450*V*
*f*_*s*2_	DC-DC converter switching frequency	15 *kHz*
*L*_*b*_	Battery side filter inductance	5 *mH*
*C*_*b*_	Battery side filter capacitance	1000 μ*F*
*V*_*b*_	Battery nominal voltage	240 *V*
*Ah*	Battery capacity	54 *Ah*

**Fig 7:**
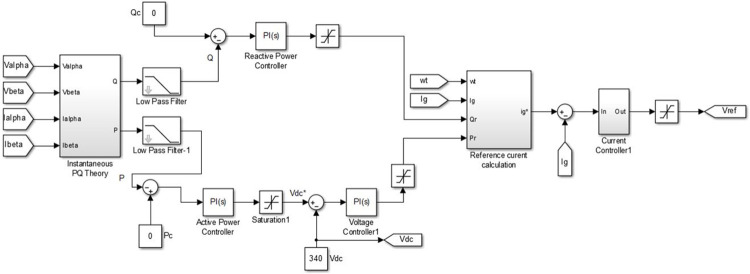
MATLAB-simulink model of controller for converter stage-1.

**Fig 8:**
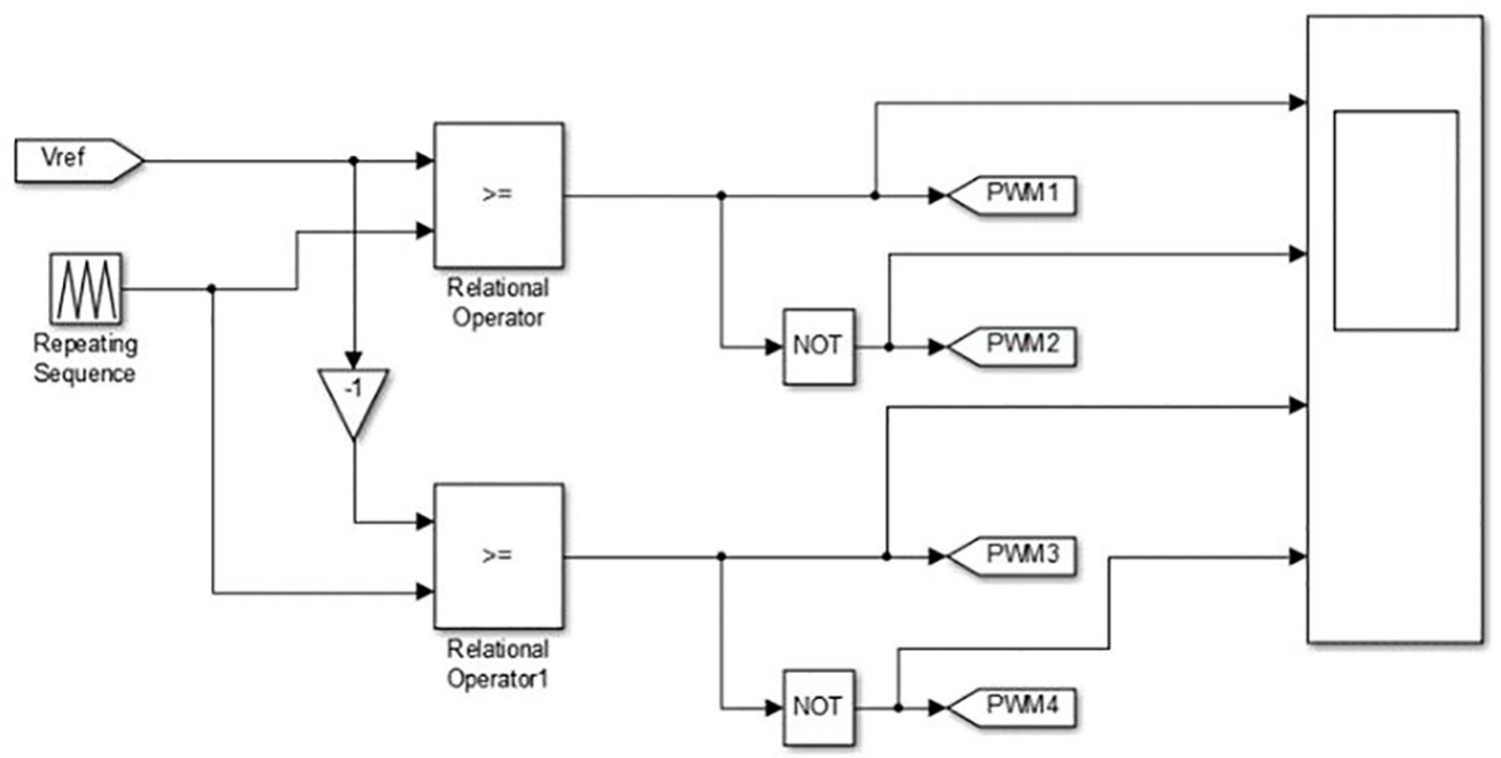
MATLAB-simulink model of bipolar PWM pulse generation.

The different modes of operation of the proposed charger are simulated by setting corresponding values for *P*_*c*_ and *Q*_*c*_ in the MATLAB Simulation model of the controller for converter stage-1depicted in Fig 7. The *V*_*ref*_ derived from the output of the controller is used to generate bipolar PWM pulses as shown in Fig 8.

**Fig 9:**
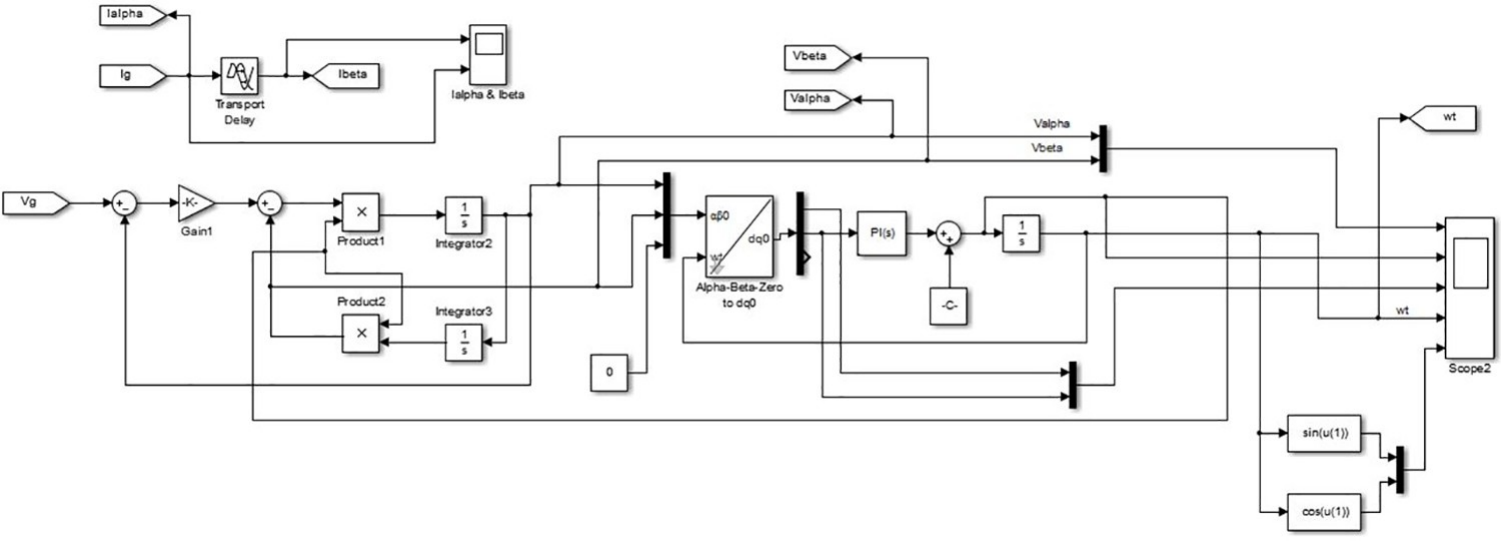
MATLAB-simulink model of SOGI-PLL.

**Fig 10:**
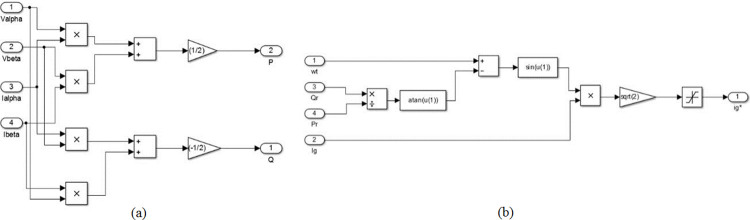
MATLAB-simulink model (a) Instantaneous PQ theory (b) Reference current calculation.

Fig 9 depicts the realization of the proposed SOGI-PLL through the basic blocks available in Simulink library. Also, the realization of instantaneous PQ theory block and reference current calculation block are described in Fig 10A and 10B respectively.

**Fig 11:**
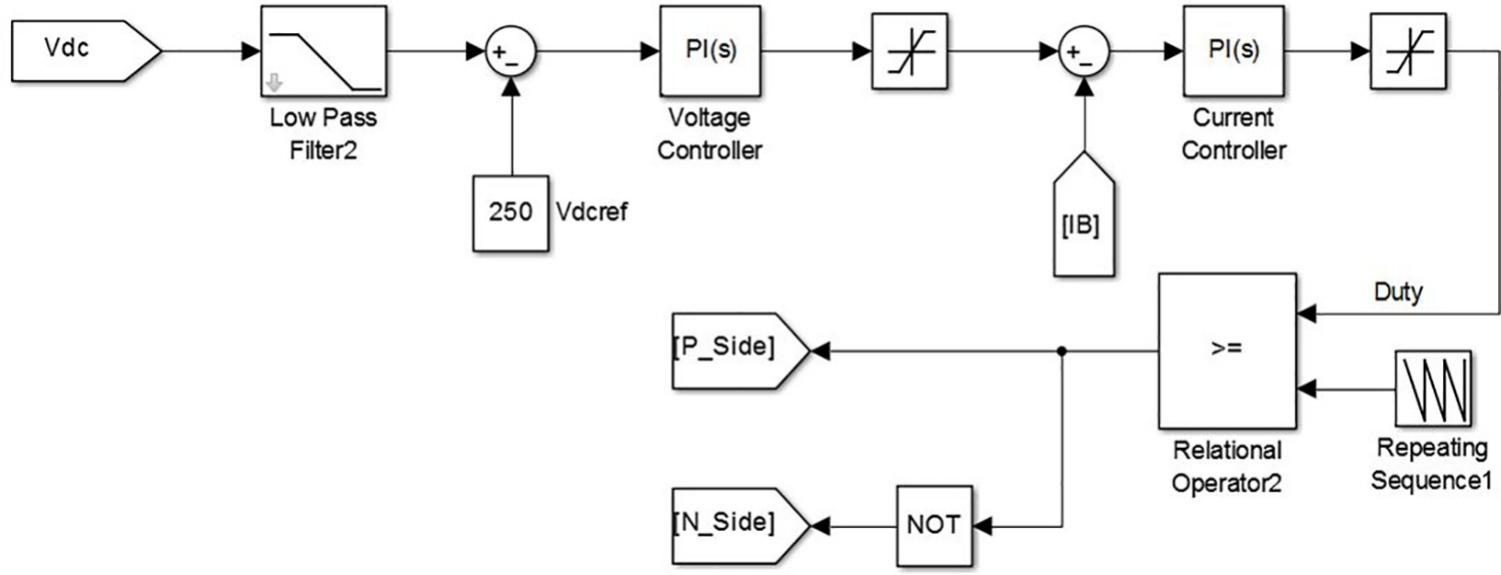
MATLAB-simulink model of controller for DC-DC converter stage.

The MATLAB-Simulink model of controller for converter stage-2 is shown in Fig 11. It is noticed that the DC link voltage is the input for this block which undergoes the transformations discussed in section III. This porcessed signal, in combination with carrier wave is used to generate the required gating pules for the swirching devices *S*_5_ and *S*_6_.

### 4.1 G2V mode of operation

Simulation starts with both *P*_*c*_ and *Q*_*c*_ set to zero during first one second to initialize the charger. This initialization is followed by three different operations during G2V mode viz. Charge-only, Charge-Capacitive and Charge-Inductive. Charge-only operation, during which the battery is charged, is simulated with *P*_*c*_ is set to 1 with *Q*_*c*_ still at zero, for next two seconds. The power factor is unity during this period. Consequent to this *P*_*c*_ and *Q*_*c*_ are set to 0.6 and -0.8 respectively to simulate reactive power requirement of grid i.e. the drop in grid voltage due to sudden increase in reactive power consumption of loads. During this Charge-Capacitive operation, the energy is supplied to grid from DC link capacitor and so the power factor is leading with respect to grid. To simulate the Charge-inductive operation i.e., functioning of the charger during a sudden increase in grid voltage due to load fluctuations, values of *P*_*c*_ and *Q*_*c*_ are set to 0.8 and 0.6 respectively. Evidently the grid power factor is lagging and the value is calculated as 0.8. Charging of battery continues during the two operations discussed above, however with reduced magnitude due to the changes in *P*_*c*_. Table 4 displays the values of all parameters during different stages of operations of the proposed charger during G2V mode.

**Table 4:** Parameters of PEV charger in G2V mode.

Type of Operation	Time Period, T	Active Power Demand	Reactive Power Demand Qc	Active Power, P	Reactive Power, Q	Apparent Power, S	Battery Charging Current, *I*_*B*_ (A)	Power Factor
				(kW)	(kVAR)	(kVA)		
	(sec)	Pc						
Initialization	0–1	0	0	0	0	0	0	-
Charge-only	1–3	1.0	0	1.35	0	1.35	5.5	1.0
Charge-Capacitive	3–5	0.6	-0.8	0.81	-1.08	1.35	3.3	0.6 (leading)
Charge-Inductive	5–7	0.8	0.6	1.08	0.81	1.35	4.1	0.8 (lagging)

### 4.2 V2G mode of operation

Table 5 shows the various parameters values observed during simulation of grid support mode. The stored energy in the battery during G2V mode of operation is utilized to deliver active power back to grid on demand, complying with grid standards. The entire V2G mode of operation is divided into three sections, viz. Discharge-only, Discharge-Capacitive and Discharge-Inductive. During Discharge-only operation, the proposed charger delivers active power to the gird at Unity Power factor (UPF). If there is a sudden fluctuation in the grid voltage, the DC link capacitor is additionally used either to deliver reactive power through Discharge-Capacitive operation or accept reactive power through Discharge-inductive operation, as demanded. As shown in Table 5, the Discharge-only operation is simulated for first two seconds of simulation by setting *Q*_*c*_ to zero. As the magnitude of *P*_*c*_ is unity, the discharging current is maximum. During next two seconds, a voltage-sag condition is simulated by setting *P*_*c*_ and *Q*_*c*_ respectively to -0.6 and -0.8 to invoke Discharge-capacitive operation. Subsequently, charger enters into Discharge-inductive operation as a voltage swell is introduced between 5 sec and 7 sec of simulation with -0.8 for *P*_*c*_ and 0.6 for *Q*_*c*_. The fall in discharging current in last two operations due to reactive action, is evident. The negative sign of the parameters indicate the power flow from PEV charger to grid in both G2V and V2G modes.

**Table 5:** Parameters of PEV charger in V2G mode.

Mode of Operation	Time Period T	Active Power Demand	Reactive Power Demand Qc	Active Power, P	Reactive Power, Q	Apparent Power S	Battery Discharging Current, *I*_*B*_ (A)	Power Factor
	(sec)	Pc		(kW)	(kVAR)	(kVA)		
Discharge-only	1–3	-1.0	0	-1.35	0	1.35	-5.5	1.0
Discharge-Capacitive	3–5	-0.6	-0.8	-0.81	-1.08	1.35	-3.3	-0.6 (leading)
Discharge-Inductive	5–7	-0.8	0.6	-1.08	0.81	1.35	-4.1	-0.8 (lagging)

## 5. Simulation results

With the architecture and the parameter values discussed in the preceding sections, the proposed charger has been simulated to evaluate its performance in both vehicle support and grid support modes of operation, under different conditions and its performance are observed. The following sections presents the performance analysis of the proposed PEV charger.

### 5.1 G2V mode

**Fig 12:**
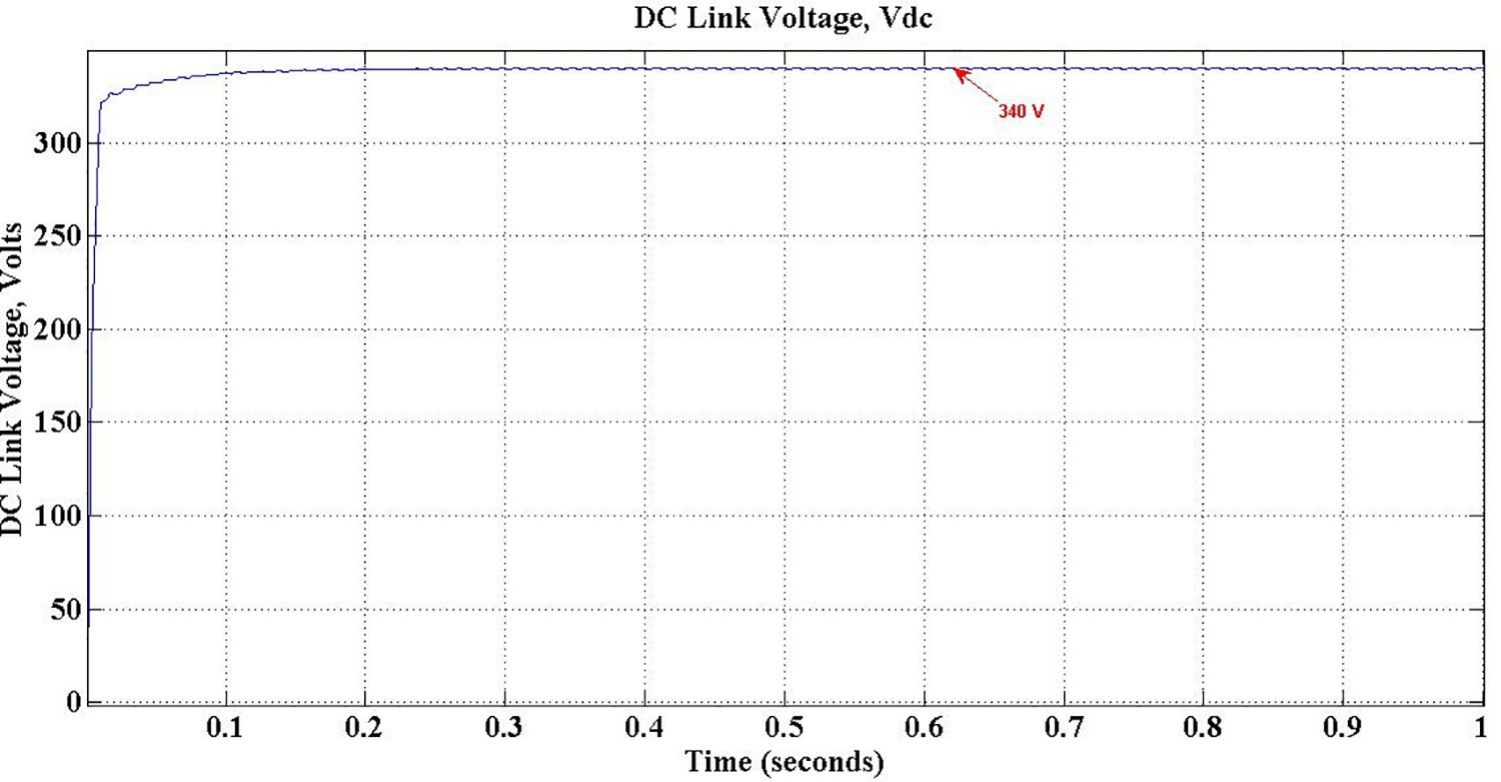
DC link voltage.

**Fig 13:**
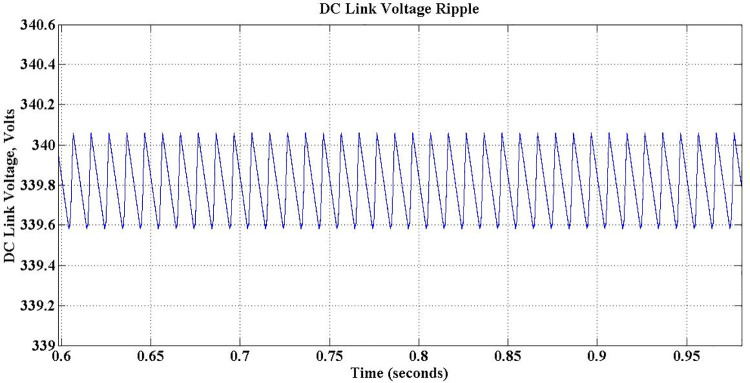
DC link voltage ripples.

Once the Charger is coupled to the grid, the voltage across DC link capacitor builds up as shown in Fig 12. The voltage reaches its desired level of 340 Volts, without any overshoot in less than 0.05 sec. Though, inevitable ripples are there during steady-state in view of switching of the devices, it is observed that the magnitude of those ripples is about 0.5 Volts, a reasonably low value. Fig 13 shows the closer look of those ripples.

**Fig 14:**
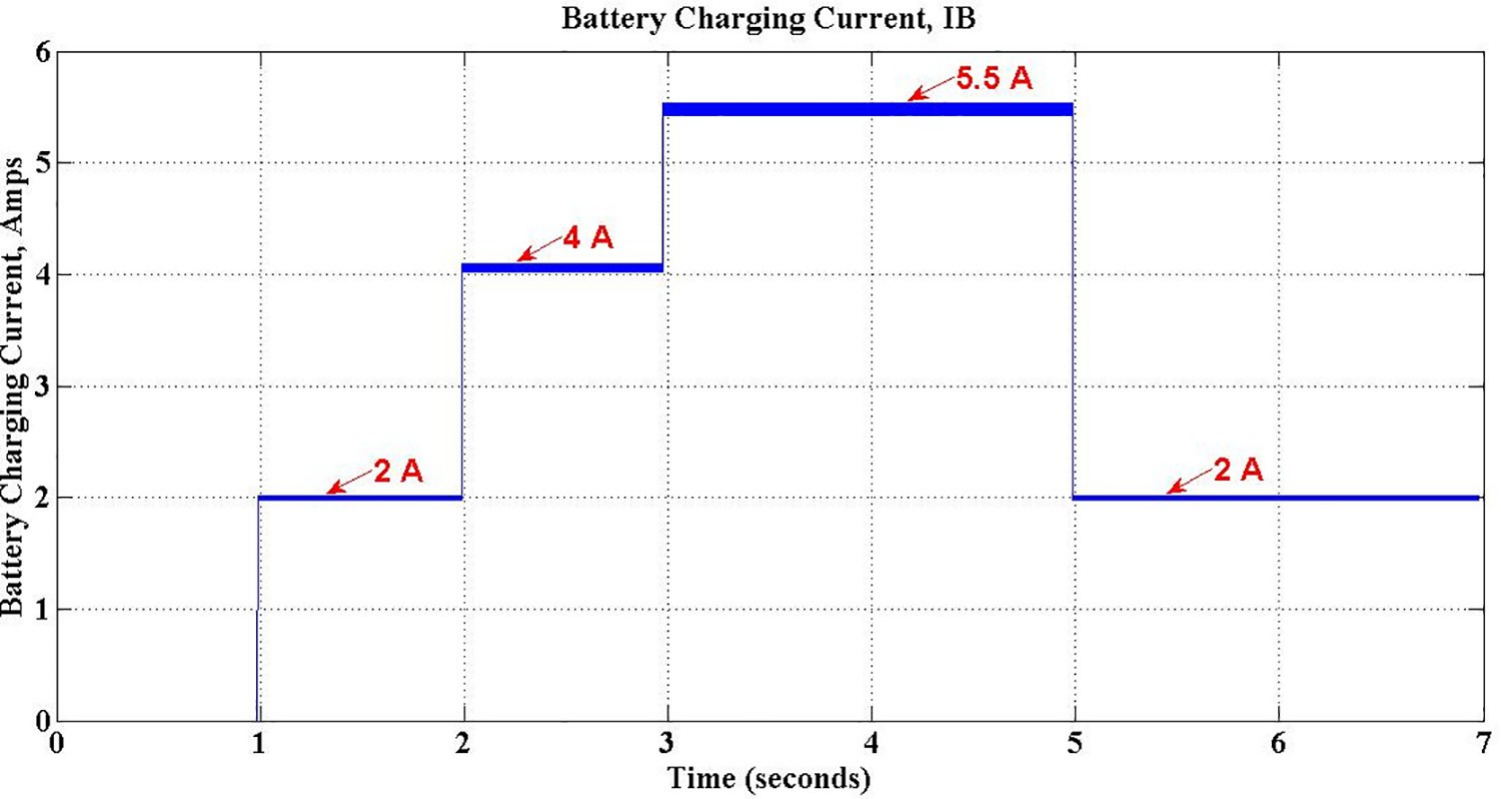
Battery charging current for different values of *P*_*c*_ during G2V mode operation.

In the proposed charger, based on the charging characteristics of the Li-Ion battery, constant current charging mode is preferred during majority of charging period of G2V Mode. In this perspective, the charging current can be set at any preferred constant magnitude by selecting appropriate value of *P*_*c*_. Fig 14 proves this process as it depicts three different desired charging currents, selected by setting *P*_*c*_ values accordingly.

**Fig 15:**
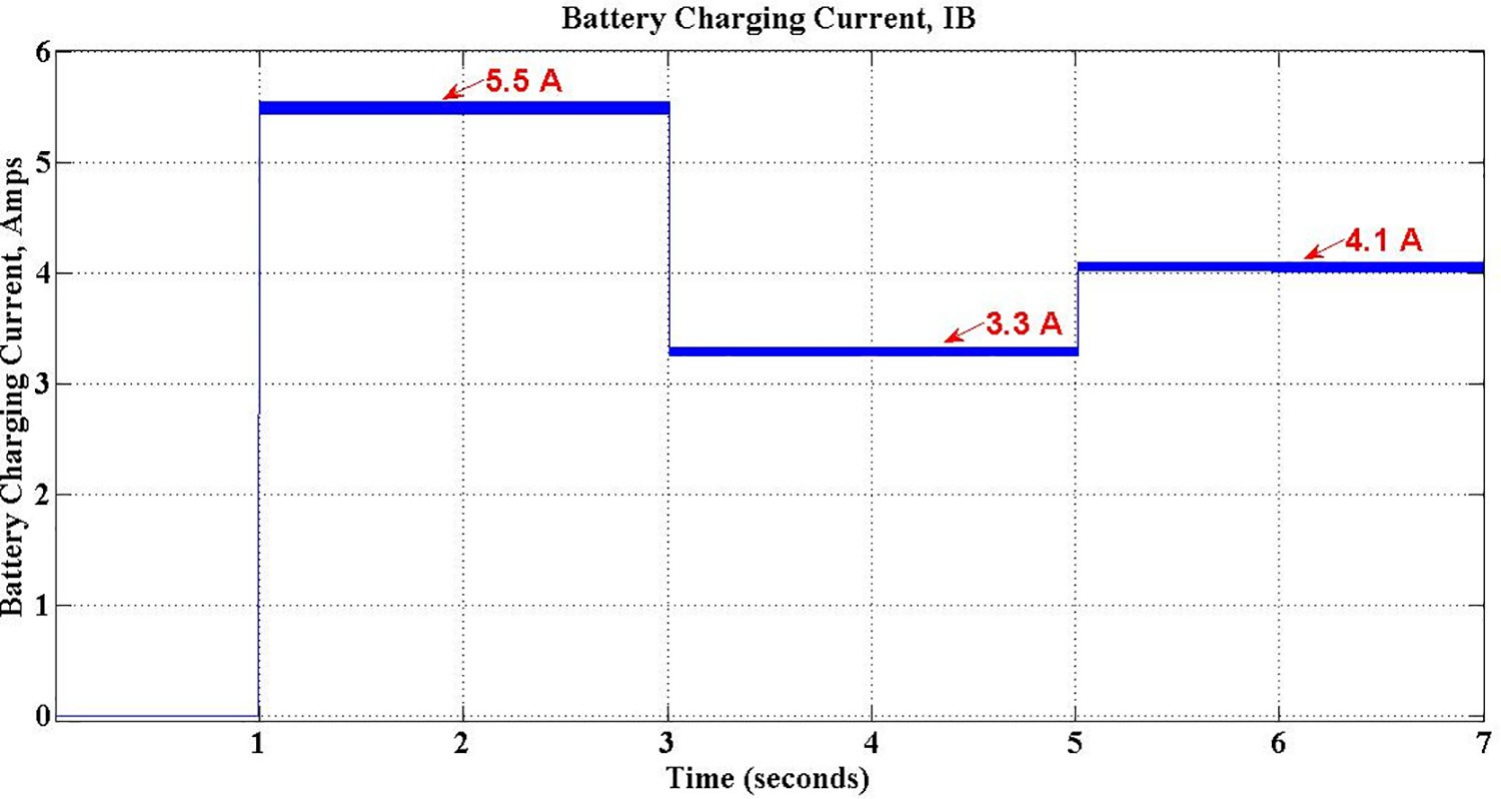
Battery charging current during G2V mode operation.

**Fig 16:**
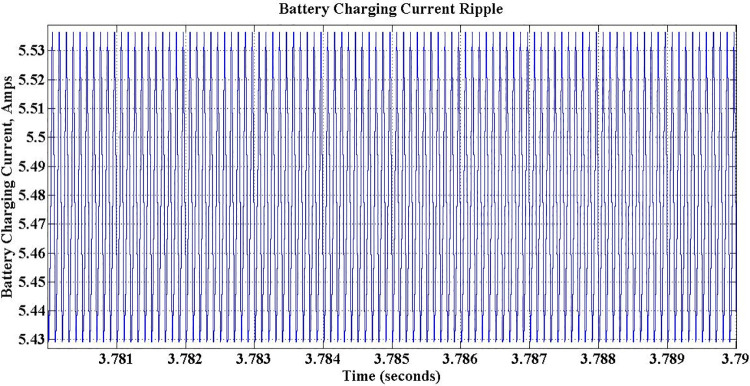
Battery charging current ripple.

Fig 15 shows the changes in the battery charging current during entire simulation period, which is initially zero for first one sec. Between 1 sec and 3 sec battery charging current is set to 5.5 Amps, the maximum value as per the design. After this period, charging current is falls down to 3.3 Amps at 3 sec and again climbs-up to 4.1 Amps at 5 sec, in accordance with the change in *P*_*c*_, to simulate two different operations of the charger. It is observed that ripples with a negligible magnitude of 0.11 Amps present in charging current also, as shown in the Fig 16.

**Fig 17:**
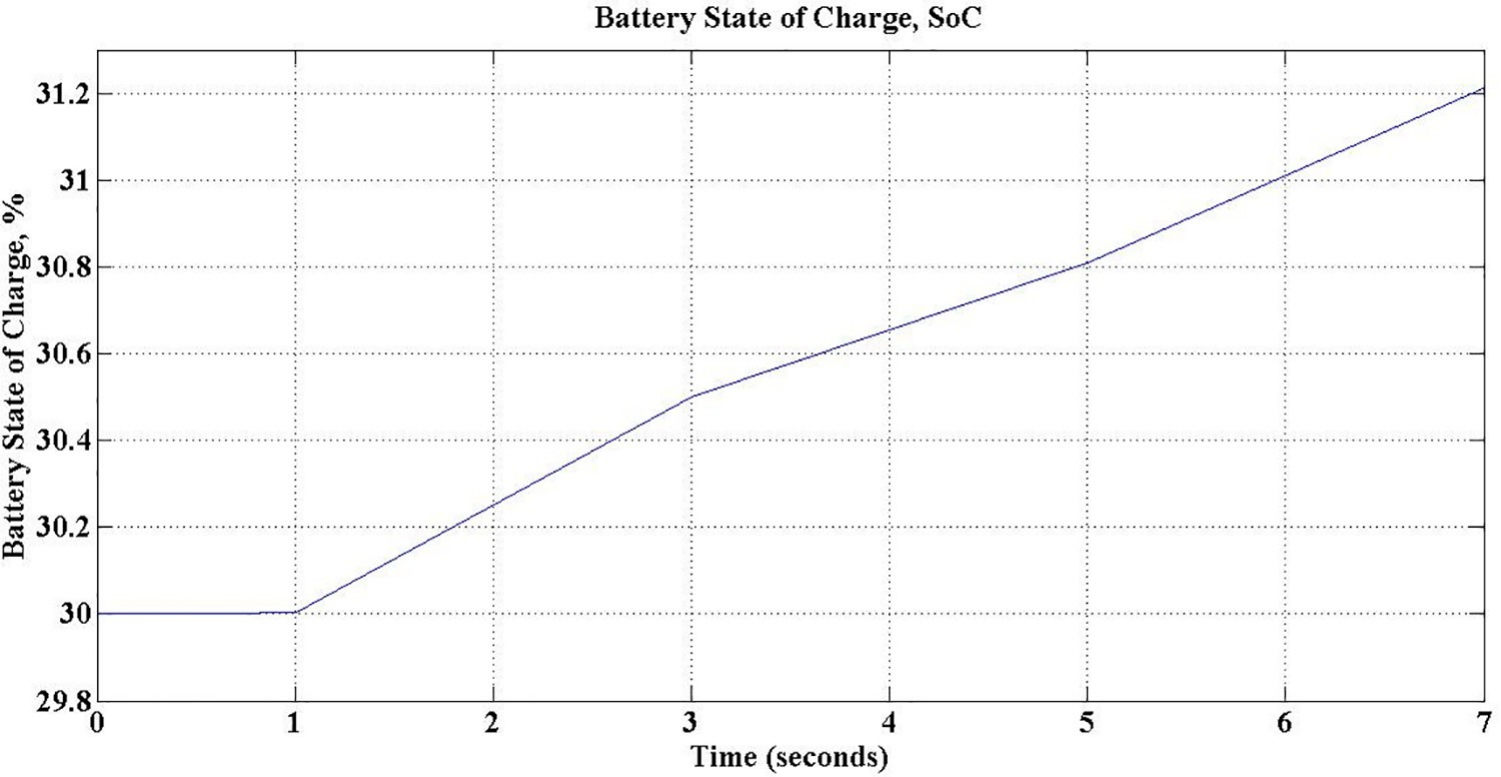
Battery state of charge (SoC) during G2V mode operation.

The battery State of charge (SoC) is set to 30% initially and Fig 17 depicts %SoC during the simulation, which ensures the charging of the battery.

**Fig 18:**
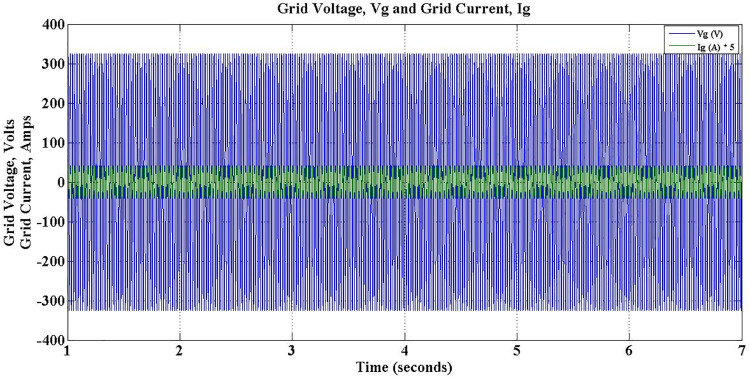
Grid voltage and grid current during G2V mode operation.

**Fig 19:**
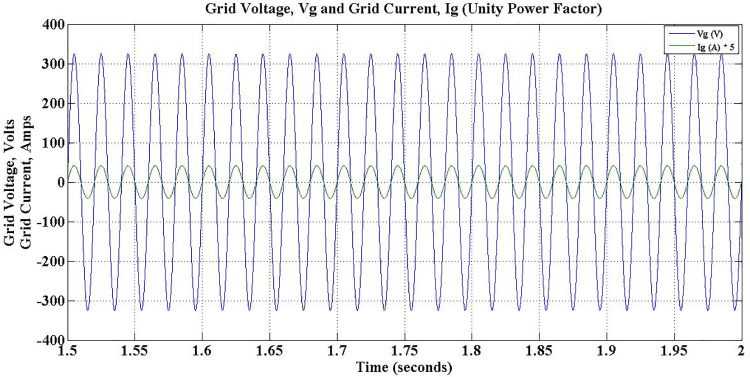
Grid voltage and grid current during G2V mode charge-only operation—unity power factor.

The grid voltage and current during different operations of the charger for the entire simulation period is depicted in Fig 18. It is evident that the charger ensures constant grid current during the entire simulation period, despite the fluctuations in load conditions. However, grid side power factor changes to reflect the direction of reactive power flow. During Charge-only operation, i.e. between 1–3 secs, the power factor is unity as shown in Fig 19.

**Fig 20:**
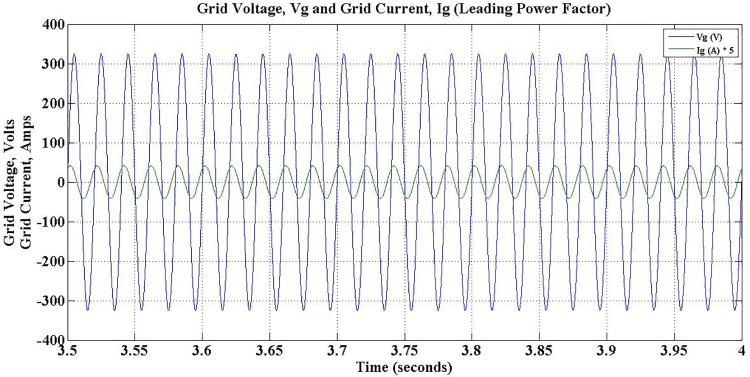
Grid voltage and grid current during G2V mode charge-capacitive operation- leading power factor.

**Fig 21:**
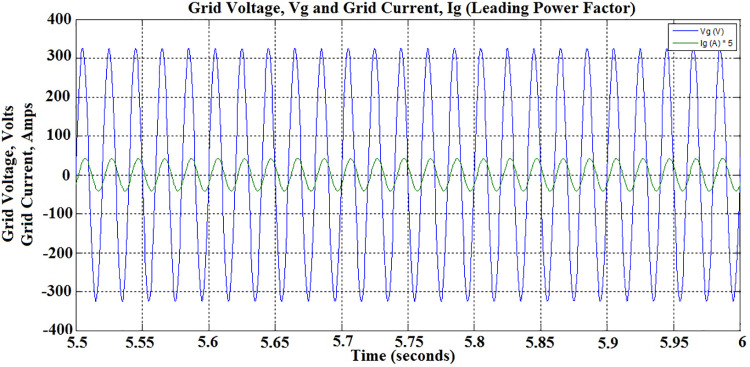
Grid voltage and grid current during G2V mode charge-inductive operation—lagging power factor.

As discussed earlier, while reactive power compensation takes place between 3–5 secs, the power factor is leading and depicted in Fig 20. Subsequently, the power factor lags for next two seconds, as the charger consumes reactive power from the grid, which is shown in Fig 21.

**Fig 22:**
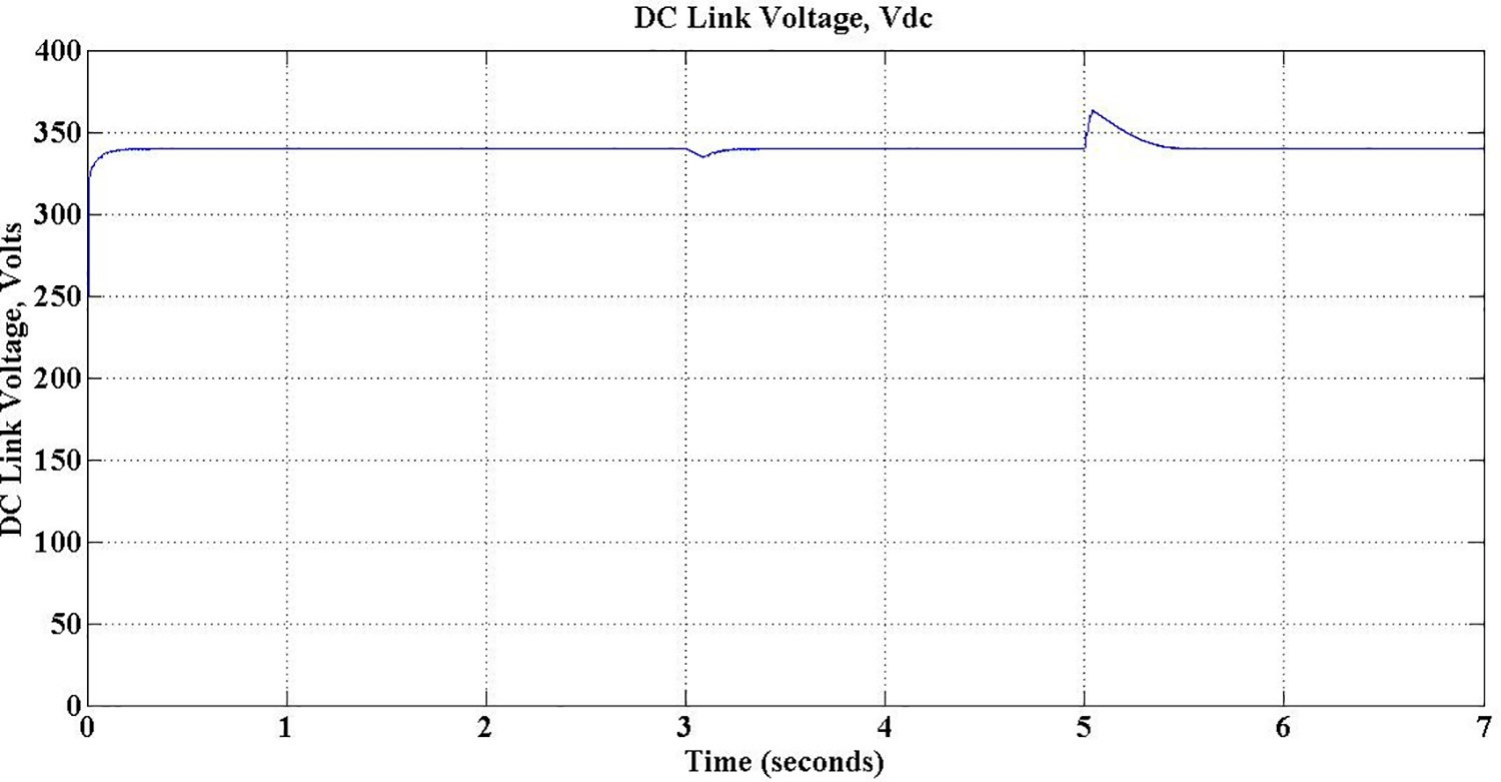
Voltage across DC link capacitor.

The reactive power stored in the DC link-capacitor is used to mitigate the voltage fluctuations in the load by delivering at lead power factor or accepting at lag power factor, as the case may be. Obviously this results in change of voltage across the DC link capacitor, indicated as voltage spikes at 3 sec and 5 sec of operation in Fig 22.

### 5.2 V2G mode

**Fig 23:**
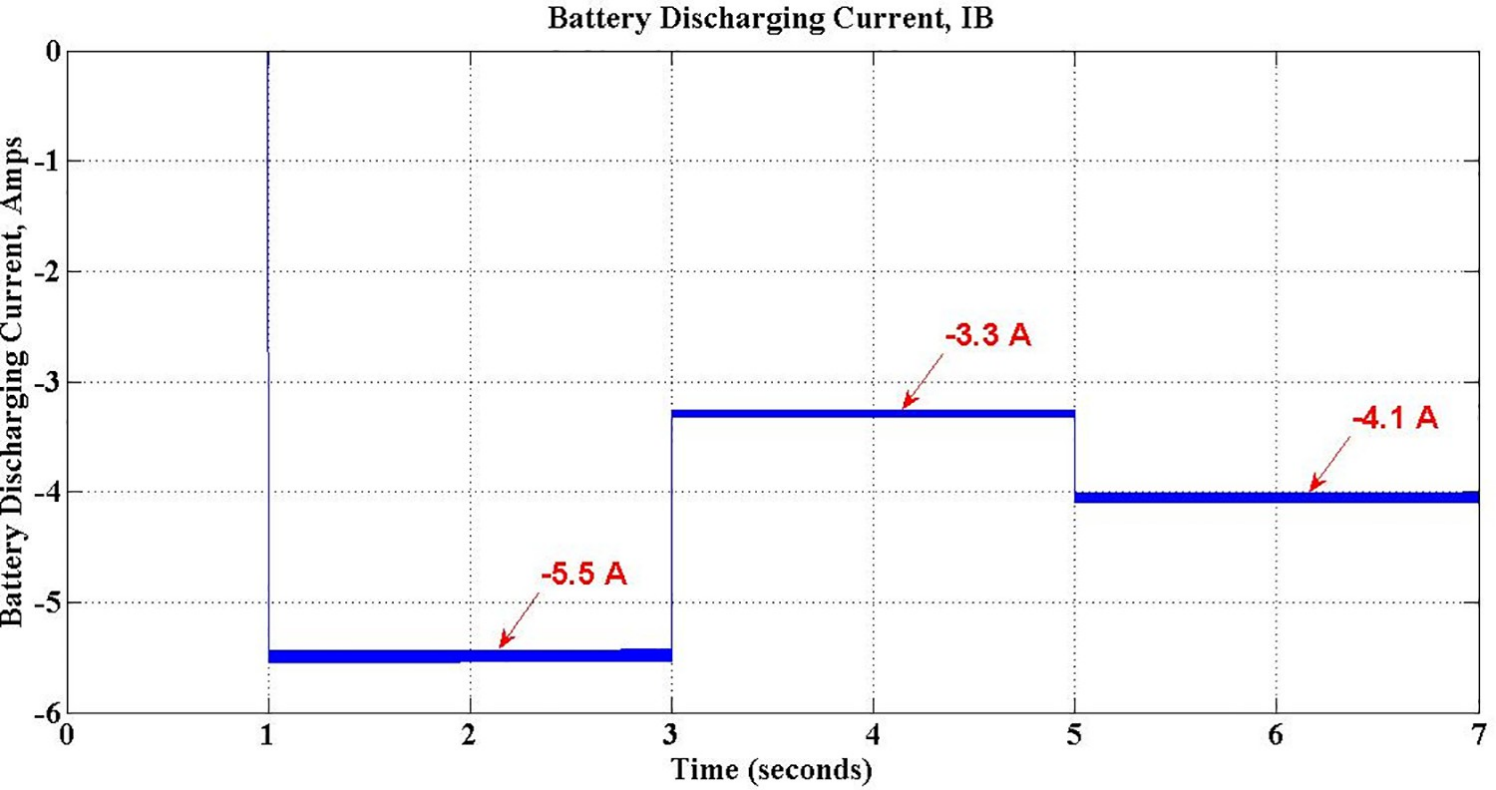
Battery discharging current during V2G mode.

**Fig 24:**
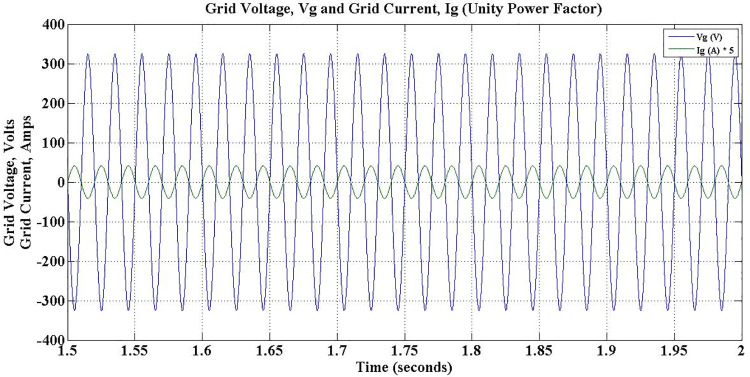
Grid voltage and grid current during V2G mode discharge-only operation—unity power factor.

The performance of the simulated PEV charger during three different operations viz. Discharge-only, Discharge-Capacitive and Discharge-Inductive are discussed in this section with relevant waveforms obtained. During Discharge-only operation, the stored energy in the battery is send to the grid, on demand, at unity power factor. This demand is simulated by setting the active demand component *P*_*c*_ to any value between 0 and 1 and *Q*_*c*_, the reactive counterpart component, to zero. This is operation is from 1 sec to 3 sec of simulation, as exposed in Fig 23. The waveforms in Fig 24 confirm the unity power-factor operation during is this period.

**Fig 25:**
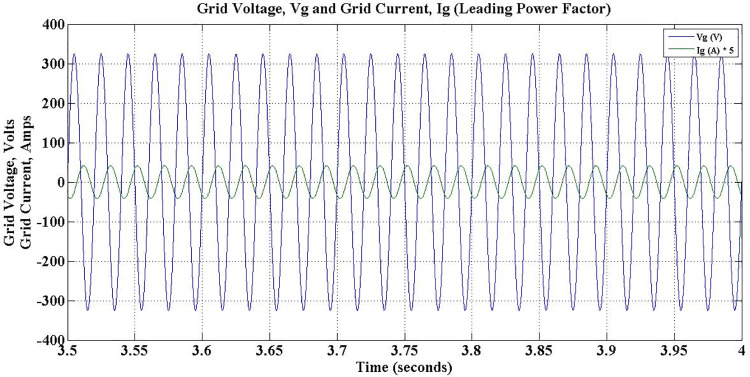
Grid voltage and grid current during V2G mode discharge-capacitive operation—leading power factor.

In addition to active power support, the proposed PEV Charger is made to support reactive power requirement of the grid during Discharge-Capacitive operation between 3 sec and 5 sec by setting *Pc* to -0.6 and *Q*_*c*_ to -0.8. Obviously, the power factor during this period is leading at 0.6and the corresponding waveforms shown in Fig 25 substantiate it. The active power demand met by battery is intact, however, with reduced current. The battery discharging current during this period is observed as -3.3 Amps as shown in Fig 23.

**Fig 26:**
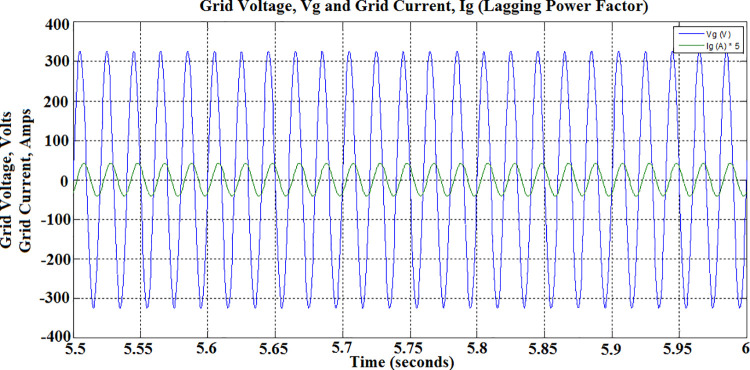
Grid voltage and grid current during V2G mode discharge-inductive operation—lagging power factor.

**Fig 27:**
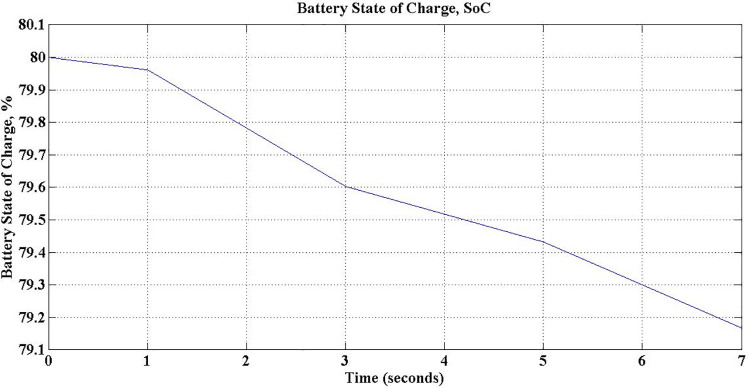
Battery state of charge (SoC) during V2G mode.

The PEV charger is so designed that it will support the grid for improved performance during unexpected voltage swells too. PEV charger absorbs the reactive power in the grid during voltage swells so as to maintain the voltage profile and evidently this can be realized only with lagging power factor operation. During 5 sec to 7 sec of simulation, the aforesaid operation is simulated with *P*_*c*_ and *Q*_*c*_ set to -0.8 and 0.6 respectively. The grid voltage and current waveforms during this period are presented in Fig 26. The reduced battery discharging current, -4.1 Amps, which is responsible for active power compensation is as depicted in Fig 23. The real power is drawn from the battery during the entire simulation of V2G mode of operations, and so, the battery SoC is obviously falling as shown in Fig 27.

**Fig 28:**
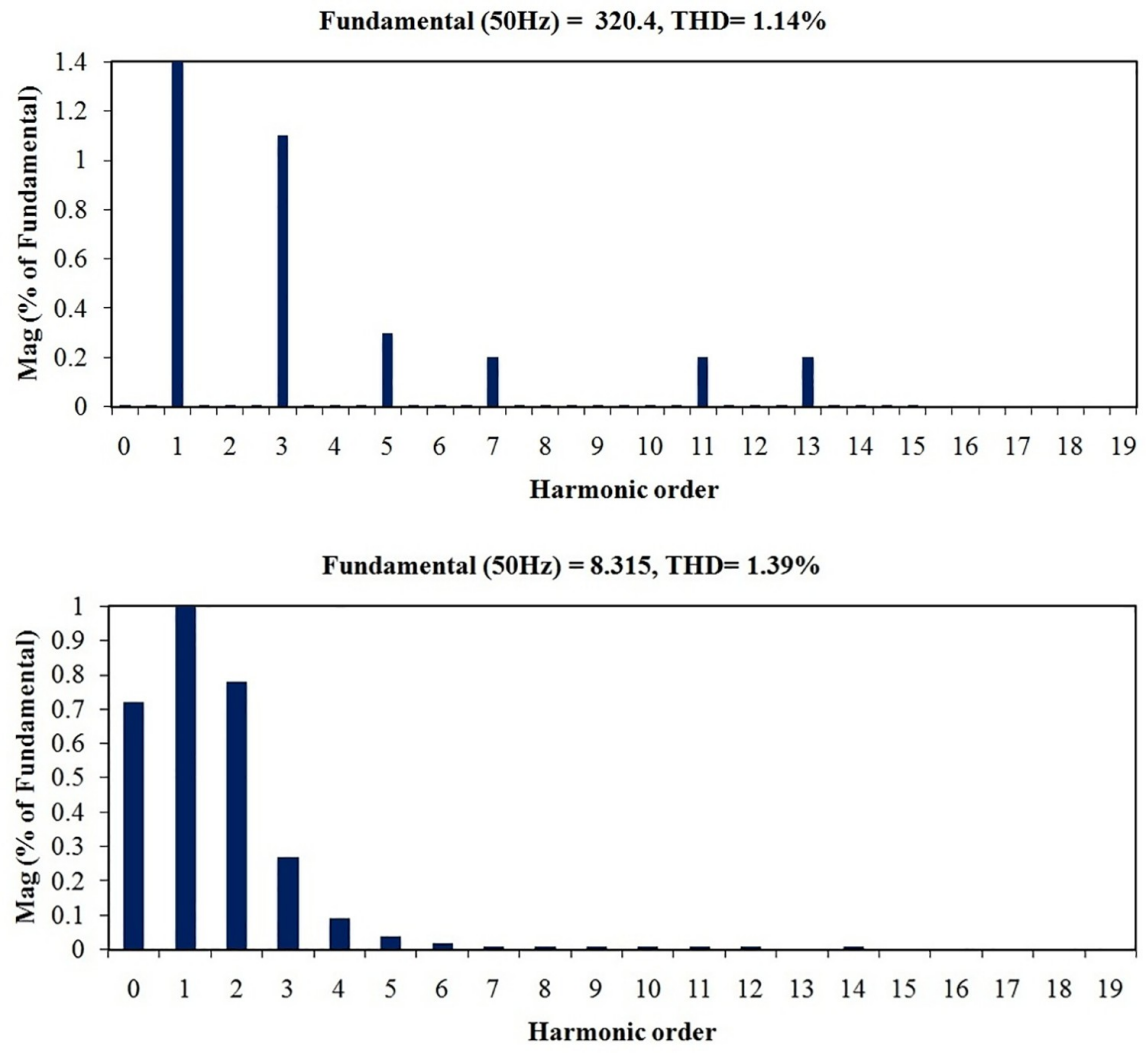
Harmonics spectrum in G2V mode of operation (a) Grid voltage (b) Grid current.

**Fig 29:**
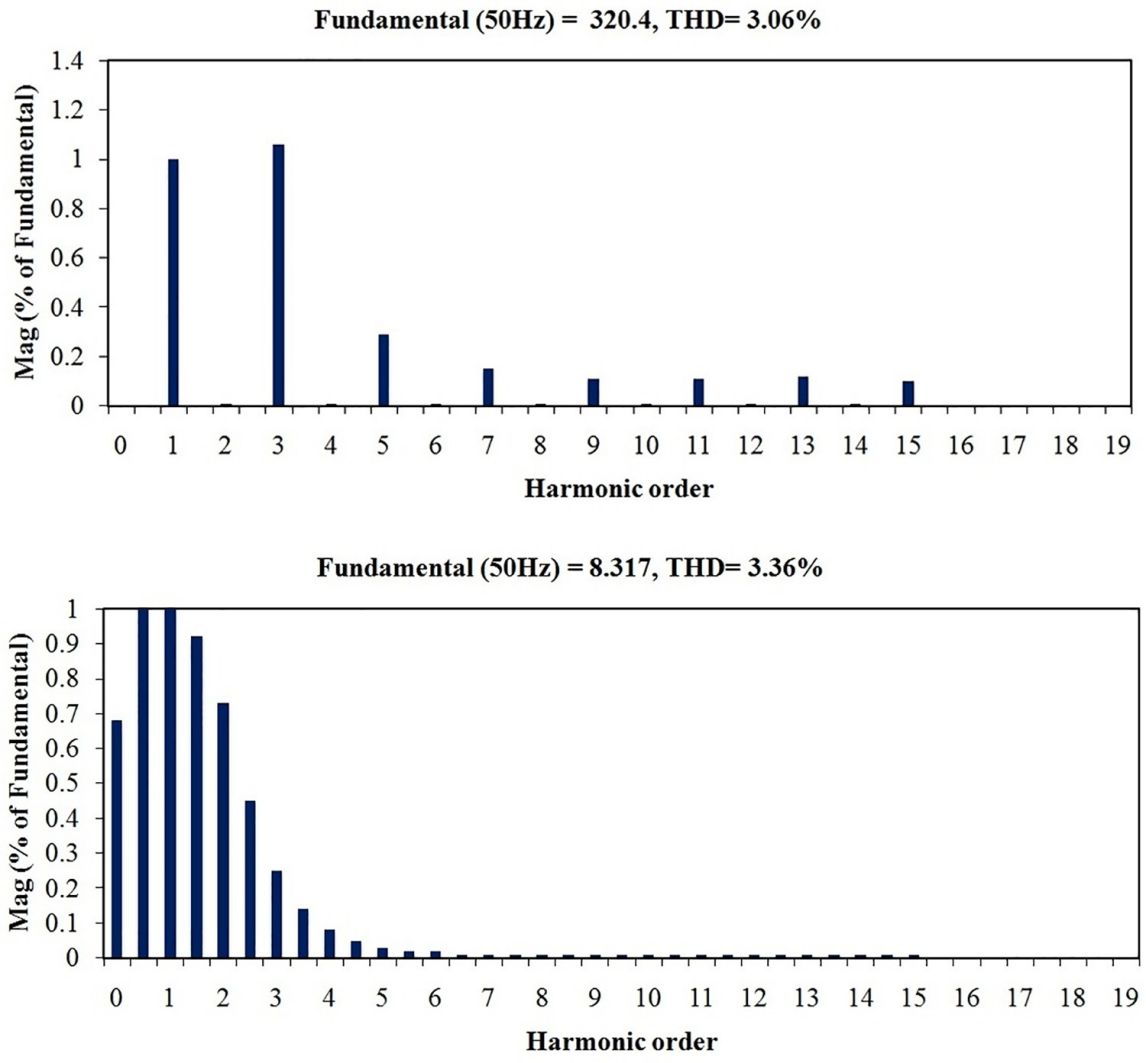
Harmonics spectrum in V2G mode of operation (a) Grid voltage (b) Grid current.

Total harmonic distortions (THD) in voltage during G2V and V2G modes of operations are projected in Figs 28A and 29A. Similarly, the respective current THDs are exposed in Figs 28B and 29B. It is noticed that the voltage THD and current THD values during G2V mode are 1.14% and 1.39% respectively. Also the same during V2G mode are 3.08% and 3.36% respectively. It is observed that all the four THD values are well below the specified IEEE and IEC standards as in [40, 41], though both current and voltage harmonics in V2G mode are relatively higher due to inverter operation.

## 6. Result and discussion

In the proposed PEV charger design, IGBTs are the switching devices whereas they are MOSFETs in the previous works [17]. Due to the structure of IGBTs, the conduction power-loss is reduced considerably in the proposed work. As shown in the comparison Table 6, the loss component is 0.067 watts per switch in the proposed PEV charger whereas the same with respect to existing model is 1.68 watts, which proves the improved power efficiency of the proposed charger. The DC link voltage overshoot is eliminated and the ripples are reduced to lower value. In the available literature, current THD in G2V mode is shown only up to 19^th^ order with 17^th^ harmonic overshooting above the specified bounds, which is a failure [17]. Conversely, in the proposed model, all harmonics above 15^th^ order have been totally eliminated, justifying the effectiveness of the latter over the former. Similarly, current THD in V2G mode is observed as 3.36%, well below the specifications, whereas it is not at all discussed for the available models.

**Table 6:** Comparison of simulation results with previous works.

S.No.	Parameter	Previous Work	Proposed Work
1	Switching Device Used	MOSFET	IGBT
2	On-state Resistance of Switching Device	Ron = 0.29 Ω	Ron = 0.0115 Ω
3	Conduction Loss per Switch	1.68 W	0.067 W
4	Power Quality Impact on Grid -Current THD	Not Discussed	1.39% @ 1.35 kW
			(>15^th^ order harmonics eliminated)
	(G2V mode)		
5	Power Quality Impact on Grid -Current THD	Not Shown	3.36% @ 1.35 kW
			(>15^th^ order harmonics eliminated)
	(V2G mode)		
6	Phase Locked Loop (PLL)	Simple PLL	SOGI PLL
7	Grid Side Filter	LC (Inrush current not shown)	LCL(No Inrush current)
8	DC Link Voltage Overshoot	Present	No Overshoot
9	DC Link Voltage Ripple	13 V to 18 V	0.5 V
10	Transient Response	0.5 sec	0.4 to 0.5 sec
11	Efficiency	92%	94%
12	Mode of Operation	G2V alone shown	Both G2V and V2G
		(2 Quadrants)	(4 Quadrants)

With SOGI PLL, the proposed PEV charger exhibits ripple magnitude of 0.5 Volt in DC link during steady-state, is a reasonably low value. This component is prohibitively higher between 13 Volt and 18 Volt in the available literature [42]. To alleviate in-rush current, LCL type filter is implemented in the proposed work, whereas a relatively simpler LC structure is used in the available models and no discussion is found on this issue in the literature [17, 18]. During charging transient, a small overshoot in DC link voltage is observed in the presently available models [17, 18], which are totally eliminated in the proposed model, though the transient period is almost same in both the cases. It is claimed in the available literature that the efficiency achieved is around 92% [17], whereas it is improved by a slender margin in the proposed model. However, only two quadrant operations is attempted in the available models [17], i.e. vehicle support mode of operation only, whereas the proposed PEV charger operates in all the four quarters of PQ plane, and so, both vehicle support and grid support modes of operation are realized. This enables the proposed system to support the grid for both active and reactive power compensation, resulting power quality improvement, a major advantage of the proposed PEV charger system comparing its available counterparts.

## 7. Conclusion

An improved version of PEV charger system of 1.35kW has been proposed in this work, with an objective to mitigate the drawbacks in the presently available system. From the literature survey it is found that the available PEV chargers have only limited grid support functionalities. Hence, to alleviate the issues found in the present chargers, a PEV charger, which can operate in both G2V and V2G modes with an ability to improve the grid performance, has been proposed. The conventional PLL used in presently available chargers has been replaced by a SOGI-PLL structure to speed-up the operation and to limit steady state oscillations. MATLAB-Simulink is used to simulate the proposed charger. Both vehicle support and grid support operation modes of the proposed charger have been simulated under different environmental conditions and the results are recorded and analyzed. The complete inference from the analysis are presented in detail. The DC link voltage and charging current ripples are significantly reduced to 0.5V and 0.11A respectively. The charger is exhibiting relatively a better performance, with an improved efficiency of 94% and reduced settling time of 0.5 sec with the proposed controller. The grid current THD, which was not considered in previous works, is observed as 3.36%, which is well within the limitations of IEEE as all harmonics above 15th order are eliminated. Also the charger offers better grid support for both voltage swells and voltage dips during both G2V and V2G modes of operation.

## References

[1] Sanguesa JA, Torres-Sanz V, Garrido P, Martinez FJ, Marquez-Barja JM. A Review on Electric Vehicles: Technologies and Challenges. Smart Cities. 2021; 4:372–404.

[2] Aguero JR, Takayesu E, Novosel D, Masiello R. Modernizing the Grid: Challenges and Opportunities for a Sustainable Future. IEEE Power and Energy Magazine. 2017; 15(3):74–83.

[3] Li Canbing, Cao Yijia, Kuang Yonghong, Zhou Bin. Influences of Electric Vehicles on Power System and Key Technologies of Vehicle-to-Grid. Berlin Heidelberg: Springer; 2016.

[4] Rogers K. M, Klump R, Khurana H, Aquino-Lugo A. A, Overbye T. J. An authenticated control framework for distributed voltage support on the smart grid. IEEE Trans. Smart Grid. 2010; 1(1):40–47.

[5] Pavić I, Capuder T, Kuzle I. Value of flexible electric vehicles in providing spinning reserve services. Appl Energy. 2015; 157:60–74.

[6] Luo Z, Hu Z, Song Y, Xu Z, Lu H. Optimal coordination of plug-in electric vehicles in power grids with cost-benefit analysis—Part i: Enabling techniques. IEEE Trans Power Syst. 2013; 28(4):3546–55.

[7] Liu H, Huang K, Yang Y. Wei H, Ma S. Real-time vehicle-to-grid control for frequency regulation with high frequency regulating signal. Prot. Control Mod. Power Syst. 2018; 3(13):1–8.

[8] Buja G, Bertoluzzo M, Chiristian Fontana. Reactive power compensation capabilities of V2G-enabled electric vehicles. IEEE Trans. Power Electronics. 2017; 32(12):9447–59.

[9] Kempton W, Letendre S E. Electric vehicles as a new power source for electric utilities. Trans. Res. Part D Transp. Environ. 1997; 2(3):157–75.

[10] Lu J, Hossain J. Vehicle-to-grid: Linking electric vehicles to the smart grid: IET Power and Energy Series; 2015.

[11] Monteiro V, Pinto JG, Afonso JL. Operation Modes for the Electric Vehicle in Smart Grids and Smart Homes: Present and Proposed Modes. IEEE Trans. Veh. Techno. 2016; 65(3):1007–20.

[12] Yilmaz Murat, Krein Philip T. Review of Battery Charger Topologies, Charging Power Levels, and Infrastructure for Plug-In Electric and Hybrid Vehicles. IEEE Trans. Power Electronics. 2013; 28(5):2151–69.

[13] Brenna Morris, Foiadelli Federica, Leone Carola, Longo Michela. Electric Vehicles Charging Technology Review and Optimal Size Estimation. Journal of Elect. Eng. & Tech. 2020; 15:2539–52.

[14] Monteiro V, Ferreira JC, Nogueiras Meléndez AA, Couto C, Afonso JL. Experimental Validation of a Novel Architecture Based on a Dual-Stage Converter for Off-Board Fast Battery Chargers of Electric Vehicles. IEEE Trans. Vehicular Tech. 2018; 67(2):1000–11.

[15] Tu Hao, Feng Hao, Srdic Srdjan, Lukic Srdjan. Extreme Fast Charging of Electric Vehicles: A Technology Overview. IEEE Trans. Transport. Electr. 2019; 5(4):861–78.

[16] Li Yang, Vilathgamuwa Mahinda, Wikner Evelina, Wei Zhongbao, Zhang Xinan, Thiringer Torbjorn, et al. Electrochemical Model-Based Fast Charging: Physical Constraint-Triggered PI Control. IEEE Trans. Ener. Conv. 2021 Mar:1–13.

[17] Kisacikoglu Mithat C., Kesler Metin, Tolbert Leon M. Single-Phase On-Board Bidirectional PEV Charger for V2G Reactive Power Operation. IEEE Trans. Smart Grid. 2015; 6(2):767–75.

[18] Melo Hugo Neves de, Trovao Joao Pedro F., Pereirinha Paulo G., Jorge Humberto M., Carlos Henggeler Antunes. A Controllable Bidirectional Battery Charger for Electric Vehicles with Vehicle-to-Grid Capability. IEEE Trans. Veh. Techno. 2018; 67(1):114–23.

[19] Gawhade Pragya, Ojha Amit. Recent advances in synchronization techniques for grid-tied PV system: A review. Energy Reports. 2021; 7:6581–99.

[20] Kulkarni A, John V. A novel design method for SOGI-PLL for minimum settling time and low unit vector distortion. Proceedings of the 5th Annual Conference of the IEEE Industrial Electronics Society; 2013 Nov 10–14; Vienna, Austria; IEEE; 2013. p. 274–79.

[21] Teodorescu Remus, Liserre Marco, Rodriguez Pedro. Grid Converters for Photovoltaic and Wind Power Systems. Wiley-IEEE Press; 2011.

[22] Prakash S, Singh JK, Behera RK, Mondal A. Comprehensive Analysis of SOGI-PLL Based Algorithms for Single-Phase System. Proceedings of the 9th National Power Electronics Conference, 2019 Dec 13–15; Tamilnadu, India; IEEE; 2019. p. 1–6.

[23] Pan Liwen and Zhang Chengning. Phase-Locked Loop Based Second Order Generalized Integrator for Electric Vehicle Single-Phase Charger. Proceedings of the 8th International Conference on Applied Energy, 2016 Oct 8–11; Beijing Shi, Cina; Elsevier; 2017. p. 4021–26.

[24] Xu Q. Qian J, Zhang B, Xie S. Harmonics and Stability Analysis of Single-Phase Grid-Connected Inverters in Distributed Power Generation Systems Considering Phase-Locked Loop Impact. IEEE Trans. Sustainable Energ. 2019; 10(3):1470–80.

[25] Verma Anjeet, Member Bhim Singh, Chandra A Kamal Al-Haddad. An Implementation of Solar PV Array Based Multifunctional EV Charger. IEEE Trans. Ind. Appl. 2020; 56(4):4166–78.

[26] Kisacikoglu Mithat C., Ozpineci Burak. EV/PHEV Bidirectional Charger Assessment for V2G Reactive Power Operation. IEEE Trans. Power Electronics. 2013; 28(12):5717–26.

[27] Jo J, Liu Z, Cha H. A New Design Method of LCL Filter for Single Phase Grid Connected Power Converter. Proceedings of International Symposium on Electrical and Electronics Engineering (ISEE); 2019 Oct 10–12; Ho Chi Minh City, Vietnam: IEEE; 2019.

[28] Villanueva Ignacio, Nimrod Vázquez Joaquín Vaquero, Claudia Hernández HéctorLópez, Osorio Rene. L vs. LCL Filter for Photovoltaic Grid-Connected Inverter: A Reliability Study. International Journal of Photoenergy. 2020; 2020:1–10. Article ID 7872916.

[29] Selvakumar RB, Vivekanandan C, Sathya E. Four-quadrant bidirectional AC-DC converter for plug-in electric vehicle charger with grid reactive power support. AIP Conference Proceedings, 2020. p. 040005-1-11. doi: 10.1063/5.0026600 33349734 PMC7750099

[30] Selvakumar RB, Vivekanandan C, Kavitha S. Two-quadrant current reversible non-isolated DC-DC converter for plug-in electric vehicle chargers. AIP Conference Proceedings, 2020. p. 040004-1-10. doi: 10.1063/5.0026600 33349734 PMC7750099

[31] Mousavi G SM, Nikdel M. Various Battery Models for Various Simulation Studies and Applications. Renewable and Sustainable Energy Reviews. 2014; 32:477–85.

[32] Tomasov Marian, Kajanova Martina, Bracinik Peter, Motyka David. Overview of Battery Models for Sustainable Power and Transport Applications. Proceedings of the 13th International Scientific Conference on Sustainable, Modern and Safe Transport; 2019 May 29–31; Novy Smokovec, Slovak Republic: Elsevier; 2019.

[33] Wei Zhongbao, Dong Guangzhong, Zhang Xinan, Pou Josep, Quan Zhongyi, He Hongwen, et al. Noise-Immune Model Identification and State-of-Charge Estimation for Lithium-Ion Battery Using Bilinear Parameterization. IEEE Trans. Industrial Elect. 2021; 68(1):312–23.

[34] Wei Zhongbao, Quan Zhongyi, Wu Jingda, Li Yang, Pou Josep, Zhong Hao, et al. Deep Deterministic Policy Gradient-DRL Enabled Multiphysics-Constrained Fast Charging of Lithium-Ion Battery. IEEE Trans. Industrial Elect. 2021 Apr:1–10.

[35] Ruan Haokai, He Hongwen, Wei Zhongbao, Quan Zhongyi, Li Yunwei. State of Health Estimation of Lithium-ion Battery Based on Constant-Voltage Charging Reconstruction. IEEE J Emerg. Selec. Topic. Power Elect. 2021 Jul:1–10.

[36] Song Datong, Sun Colin, Wang Qianpu, Jang Darren. A Generic Battery Model and Its Parameter Identification. Energy and Power Engineering. 2018; 10:10–27.

[37] mathworks.com [Internet]. Generic battery model, MATLAB R2021a; 2021. Available form: https://in.mathworks.com/help/physmod/sps/powersys/ref/battery.html

[38] Timbus Adrian, Liserre Marco, Teodorescu Remus, Rodriguez Pedro, FredeBlaabjerg. Evaluation of Current Controllers for Distributed Power Generation Systems. IEEE Trans. Power Elect. 2009; 24(3):654–64.

[39] Kisacikoglu MC, Abdulkadir Bedir, Ozpineci B, Tolbert LM (Oak Ridge National laboratory). PHEV-EV charger technology assessment with an emphasis on V2G operation. Office of Scientific and Technical Information (US), Oak Ridge National Laboratory; 2012 Mar. Report No.: ORNL/TM-2010/221. Contract No.:DE-AC05-00OR22725.

[40] IEEE Recommended Practices and Requirements for Harmonic Control in Electrical Power Systems, IEEE Standard. 519–1992 (2002).

[41] Electromagnetic Compatibility EMC—Part 3–2: Limits—Limits for Harmonic Current Emissions (Equipment Input Current ≤ 16 A Per Phase), IEC Standard 61000-3-2 (2005).

[42] Kisacikoglu MC, Ozpineci B, Tolbert LM. Examination of a PHEV bidirectional charger system for V2G reactive power compensation. IEEE Applied Power Electronics Conference and Exposition, 2010. p. 458–65.

